# Cationic Amino Acids Specific Biomimetic Silicification in Ionic Liquid: A Quest to Understand the Formation of 3-D Structures in Diatoms

**DOI:** 10.1371/journal.pone.0017707

**Published:** 2011-03-03

**Authors:** Rajesh Ramanathan, Jos L. Campbell, Sarvesh K. Soni, Suresh K. Bhargava, Vipul Bansal

**Affiliations:** School of Applied Sciences, RMIT University, Melbourne, Victoria, Australia; University of Helsinki, Finland

## Abstract

The intricate, hierarchical, highly reproducible, and exquisite biosilica structures formed by diatoms have generated great interest to understand biosilicification processes in nature. This curiosity is driven by the quest of researchers to understand nature's complexity, which might enable reproducing these elegant natural diatomaceous structures in our laboratories via biomimetics, which is currently beyond the capabilities of material scientists. To this end, significant understanding of the biomolecules involved in biosilicification has been gained, wherein cationic peptides and proteins are found to play a key role in the formation of these exquisite structures. Although biochemical factors responsible for silica formation in diatoms have been studied for decades, the challenge to mimic biosilica structures similar to those synthesized by diatoms in their natural habitats has not hitherto been successful. This has led to an increasingly interesting debate that physico-chemical environment surrounding diatoms might play an additional critical role towards the control of diatom morphologies. The current study demonstrates this proof of concept by using cationic amino acids as catalyst/template/scaffold towards attaining diatom-like silica morphologies under biomimetic conditions in ionic liquids.

## Introduction

Silica is an important inorganic material due to its extensive use in a wide range of applications including molecular sieves, resins, catalysts, polymer support, and biomedicine. Chemical synthesis of silica-based materials is well established, but often requires high temperatures, pressure, and pH [1]. In contrast, biosilicification in living organisms such as cyanobacteria, diatoms, sponges, and plants proceeds under mild physiological conditions that result in complex and hierarchical silica nanostructural frameworks with exquisite morphologies [2]–[6]. During biosilicification under natural marine conditions, diatoms and sponges take up silicon in the form of silicic acid from sea water, and catalyze its polymerization through diffusion-limited precipitation of silica [7] to form well-defined ornate biogenic silica structures in their skeletons [8]–[12]. The biosilicification process in these organisms has been extensively studied, and is hitherto believed to be triggered via hydrolysis of silicic acid by cationic proteins such as silicateins [8]–[15] and polycationic peptides such as silaffins [8]–[13], [16], [17]. These biomolecules are believed to act as catalyst/template/scaffold during biosilica formation, and in vitro studies involving biomolecules have demonstrated biosilica formation under biomimetic laboratory conditions [8]–[17]. Similarly, our previous work has also extensively investigated the synthesis of biosilica and other oxide nanomaterials using microorganisms such as fungi [2], [18]–[23]. It is noteworthy that biosilicification in these living organisms lead to formation of exquisite, intricate, and most often hierarchical and symmetric patterns of amorphous biosilica nanostructures, which are generally species-specific, and genetically controlled [24]. Notably, apart from the aesthetics of biogenic silica constructions in nature, a control over silica morphology under laboratory conditions is desirable, as it will inspire us to understand this complicated process, and in turn will provide a pathway for large scale fabrication of these biomaterials for various applications [25]–[27].

Since biosilicification by living organisms mostly employs aqueous environments in their natural habitats, water has so far been considered as the most obvious medium of choice to study biomimetic silicification under laboratory conditions. In fact, to the best of our knowledge, most of the biomimetic silicification studies reported thus far in the literature have employed aqueous solutions. Despite significant efforts towards understanding the biosilicification processes, as far as the control over silica morphology is concerned, to the best of our knowledge, most of the previous studies have hitherto not been able to produce 3D ornate silica morphologies through biomimetic silicification, similar to those produced by diatoms and sponges in their natural habitats [28]. Off late, it has been recognized that in addition to biomolecules involved in biosilicification, the physico-chemical environment in which this process happens in these organisms may also play a major role towards dictating the shape of nanostructured silica in these organisms [28]. The role of these physico-chemical parameters such as how silicic acid is taken up from the surrounding marine environment of diatoms, transported to silica deposition vesicles (SDVs), deposited in 1000-fold higher than environmental amount in SDVs, and further condensed into ornate silica structures still remains unclear [28]. Similarly, the effects of high salt concentrations, various physico-chemical forces, and high pressure levels including shear stress under deep marine conditions where these organisms grow, is also a mystery. Notably, in one of the previous studies, when external forces such as electrostatic field or shear stress was applied during biomimetic synthesis of silica in water, fused silica nanoparticles in the form of fibers, platelets and dendritic silica could be obtained [29]. However, the possibility to achieve ornate hierarchical nanostructures that resemble diatom frustules or sponge spicules is still out of reach [12], [28]. This also suggests that the previous biomimetic studies, which were thus far explored in aqueous solvents [8]–[17], do not necessarily mimic the natural biosilicification environment.

Ionic liquids - ILs (commonly referred as room temperature molten salts) - have recently attracted significant attention as a promising new class of environmentally-benign ‘green’ solvents for nanomaterials synthesis due to their unique physico-chemical properties such as high viscosity, high vapour pressure, high ionic conductivity, high thermal and chemical stability, and negligible volatility [30], [31]. Unlike conventional aqueous solutions, IL solvents can dissociate into individual cations and anions rather than existing as intact molecules, and due to their molten salt nature at room temperatures [32], ILs can in principle also mimic supersaturated salt solutions under marine conditions where biosilicification process takes place. Moreover, we recently demonstrated that ILs can promote non-equilibrium large-order assembly of metal and metal oxide nanoparticles through a diffusion-limited aggregation (DLA) process [33]–[35], similar to that, as believed to be involved in diatom biosilica formation [7], [36]. This suggests that such properties of ILs may in turn provide a control over biomimetic silicification. Although, the potential of ILs has just begun to play an important role in biosciences as one of the most interesting areas, with applications in enzyme stabilization, protein crystallization, and bio-fuel cells [30], to the best of our knowledge, the potential of ILs as designer solvents for performing biomimetic reactions has not so far been explored, more so, in the context of biosilicification. To this end, we envisage that replacing conventional non-ionic solvents with ionic solvents such as ILs during biomimetics might significantly influence the reaction outcomes due to unique properties of these solvents, which might provide a control over biomimetic silicification, leading to unique silica structures.

In the current study, we demonstrate for the first time that well-defined silica structures that seem to resemble the morphology of diatoms present in nature to some extent, can be synthesized at room temperature by using IL 1-butyl-3-methylimidazolium-tetrafluoroborate [BMIM][BF_4_] as a unique solvent for biomimetic silicification, and cationic amino acids as bio-catalysts. We believe that this first report on biomimetic silicification in IL will fuel significant interest in exploring biomimetic reaction in ILs.

## Results and Discussion

In a typical synthesis, 1 mM tetraethylorthosilicate (TEOS) was independently exposed to three key cationic amino acids (L-lysine, L-arginine and L-histidine) in IL [BMIM][BF_4_] at 25±0.1°C under non-stirring condition for 16 h (see experimental details). Three different molar ratios of TEOS to amino acid viz. 1∶10, 1∶1 and 1∶0.2 were used to validate the influence of amino acids as shape-directing templates. In [BMIM][BF_4_], all the reactions involving amino acids and TEOS became turbid, suggesting the hydrolysis of TEOS by cationic amino acids in IL, following which the reaction products were analysed using scanning electron microscopy (SEM), X-ray diffraction (XRD), selected area electron diffraction (SAED), and X-ray photoemission spectroscopy (XPS). However, in a control experiment, wherein IL was replaced with deionized water in the presence of amino acids, no turbidity or precipitation was observed, thereby negating the possibility of water-mediated hydrolysis of TEOS in [BMIM][BF_4_]. This also suggests that cationic amino acids serve as effective promoters for spontaneous precipitation of silica via TEOS hydrolysis in [BMIM][BF_4_], but the same amino acids do not cause TEOS hydrolysis in deionized water.

**Figure 1:**
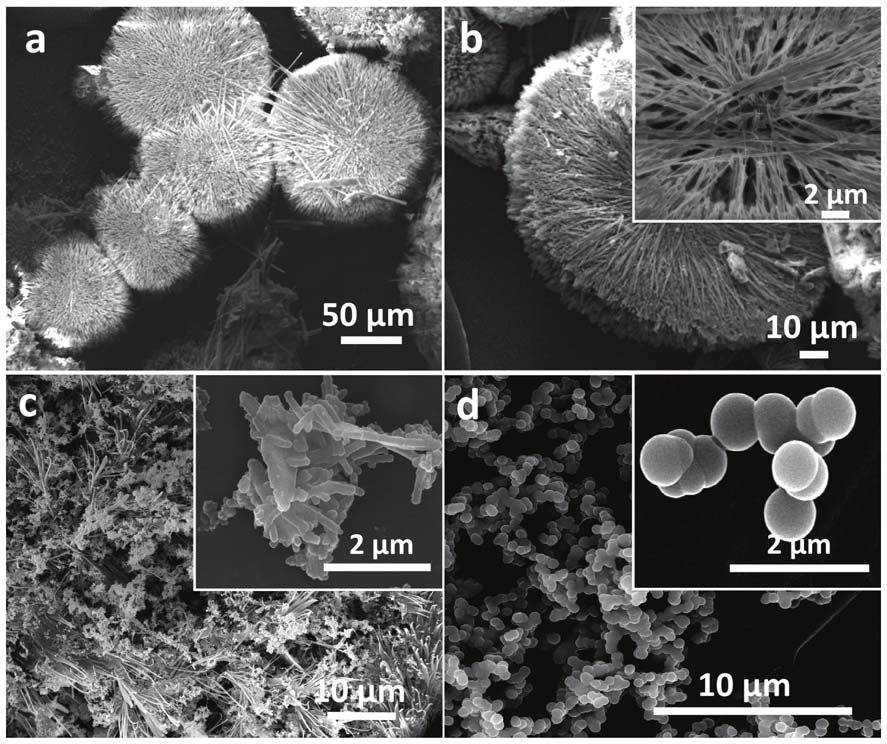
SEM images of silica structures synthesized using lysine in IL [BMIM][BF_4_] involving TEOS to lysine molar ratios of (a and b) 1∶10, (c) 1∶1, and (d) 1∶0.2 respectively. The insets show the higher magnification images of the structures shown in the corresponding main figure.

**Figure 2:**
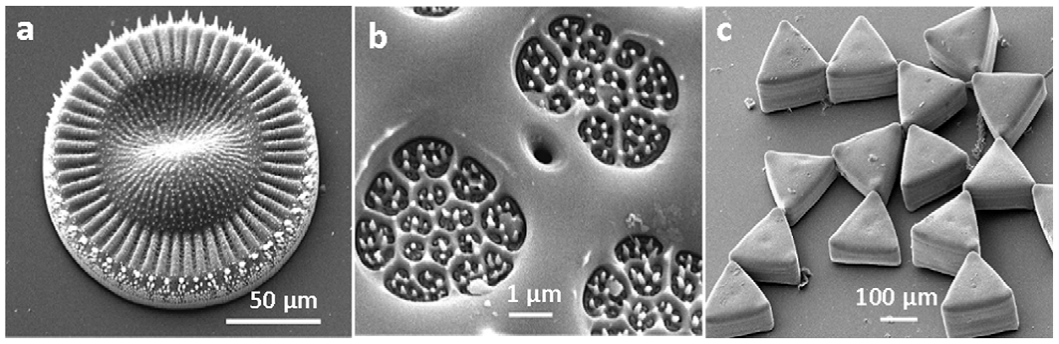
SEM images of found in nature that have some resemblance to silica structures synthesized by cationic amino acid in ionic liquid [BMIM][BF_4_]. (a) *Isthima nervosa*, (b) *Cyclotella meneghiniana*, and (c) *Trigonium arcticum*. Reproduced by permission from *Trends in Biotechnology* [Drum R and Gordon R (2003) Star Trek replicators and diatom nanotechnology. *Trends Biotechnol* 21: 325–328]

Figure 1 shows the representative SEM images of silica structures obtained in [BMIM][BF_4_] while employing TEOS to lysine molar ratios of 1∶10 (a–b), 1∶1 (c) and 1∶0.2 (d) respectively. At higher lysine concentration (10 mM), well-defined silica microdiscs of 75–150 µm in diameter with brush-like appearance were formed (Figure 1a–b). On closer observation, one of the microdiscs lying tilted on the surface of SEM substrate reveals that these silica microdiscs are about 10 µm in thickness (Figure 1b). A higher magnification of the image (inset, Figure 1b) further indicates that these microdisc-like structures are formed in IL in the presence of lysine via assembly of 75–150 µm long and 50–200 nm thick silica nanorods. It is notable that although lysine and other cationic amino acids are believed to be involved in silicic acid hydrolysis during biosilicification, these amino acids stand alone were found not to hydrolyse TEOS in water under biomimetic conditions [12]. This is predominantly because polymers of these cationic amino acids such as polycationic peptides and proteins are generally considered to be essential for promoting TEOS hydrolysis in aqueous solutions [37]. TEOS hydrolysis using lysine in IL [BMIM][BF_4_] is therefore rather interesting and suggests that IL might play a role in self assembly/organization of lysine molecules into polycationic amines like physical environment, thus promoting TEOS hydrolysis. Even more intriguing is the observation that silica structures formed by lysine-mediated hydrolysis in IL seem to have some resemblance with the overall complex silica matrices formed by few diatom species (e.g. *Isthmia nervosa*) during biosilicification in nature (Figure 2a) [7]. Notably, diatoms being biological entities, have intricate biosilica architectures in terms of well-defined overall morphology, shape, size, surface patterns and hierarchical porosity, which results from a complex interplay of a range of genetic and environmental factors within an organic matrix. Therefore, it will probably be one of the most difficult research endeavours to mimic their forms completely. To this end, it must also be noted that the silica superstructures formed by lysine-mediated TEOS hydrolysis in IL do not entirely resemble with the diatom species *I. nervosa* in all the aforementioned parameters, but seems to bear resemblance in overall size and shape of these structures. Nevertheless, this is for the first time that ensembles of silica nanoparticles formed via biomimetic silicification have led to superstructures with a degree of resemblance to those formed by diatoms in natural habitats. To further investigate the role of cationic amino acid lysine towards formation of these ornate structures, lysine concentration was reduced to 1 mM while maintaining the ratio of TEOS to lysine molecules as 1∶1 and 1∶0.2 respectively in two separate experiments. It is evident from Figure 1c that although silica rods are still formed at 1∶1 TEOS to lysine ratio, these structures are less well-defined, and they do not assemble to form silica micro-discs. A further shift in TEOS over lysine ratio to 1∶0.2 results in complete loss of morphology control, thereby mostly leading to silica spheres of ca. 700 nm diameter (Figure 1d). This provides a clear evidence that lysine molecules provide a shape-directing effect during biomimetic silicification in IL [BMIM][BF_4_].

**Figure 3:**
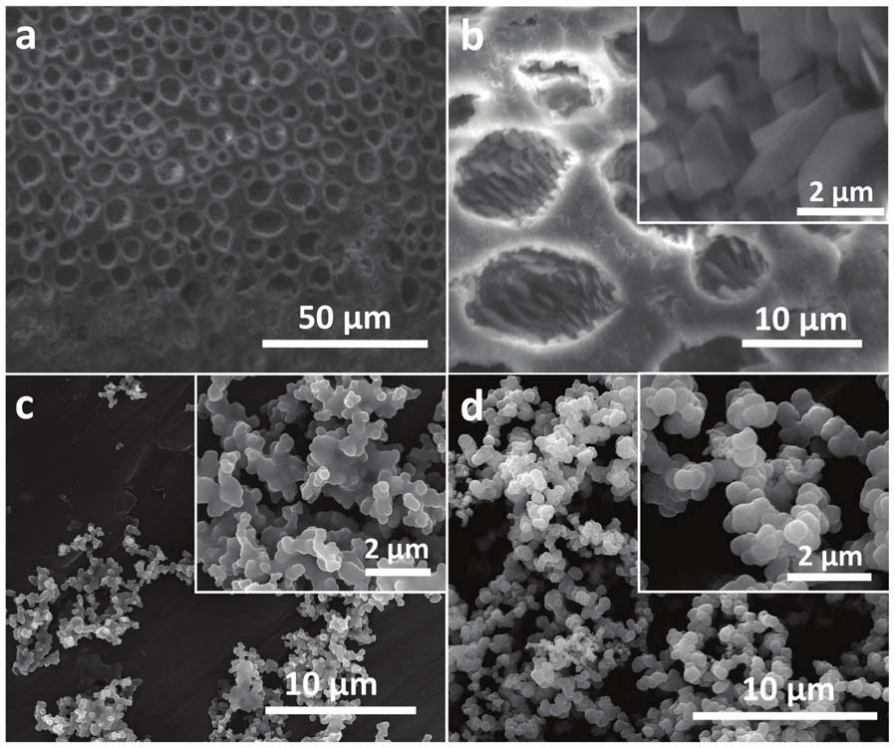
SEM images of silica structures synthesized using arginine in IL [BMIM][BF_4_] involving TEOS to arginine molar ratios of (a and b) 1∶10, (c) 1∶1, and (d) 1∶0.2 respectively. The insets show the higher magnification images of the structures shown in the corresponding main figure.

In addition to lysine, two other cationic amino acids viz. arginine and histidine are also known to be involved in biosilicification processes [28], [38]. We, therefore further investigated arginine- and histidine-mediated biomimetic silicification in IL [BMIM][BF_4_] to explore whether structural control via biomimetic silicification in IL is an amino acid-specific feature, as is observed in diatoms. When arginine was employed for TEOS hydrolysis in [BMIM][BF_4_] at 1∶10 TEOS to arginine ratio, long extended porous sheet-like silica structures with 5–10 µm pore diameter were obtained (Figure 3a–b). At higher magnification, these micropores were foundto be filled with closely-packed rectangular platelets of 1–2 µm edge length (Figure 3b). Notably, biosilica structures formed by a large number of diatom species are well-known to possess hierarchical porosity within their silica architectures [7]. For instance, the porous surface obtained by arginine-mediated hydrolysis of TEOS in IL has some resemblance in terms of overall pore size and pore distribution pattern with that of diatom species *Cyclotella meneghiniana* (Figure 2b) [7]. However, this similarity is still distantly far from the level of hierarchical porosity demonstrated by diatomaceous silica in the natural environments. Similar to lysine system, when arginine concentration was reduced to 1 mM (1∶1 ratio, Figure 3c) or 0.2 mM (1∶0.2 ratio, Figure 3d), assembled networks of ca. 400 nm silica particles, were formed. Formation of long range ordered porous silica morphologies at higher arginine concentrations, and their absence at lower arginine concentrations clearly suggests the shape-directing effect of arginine molecules during biomimetic silicification in IL [BMIM][BF_4_].

**Figure 4:**
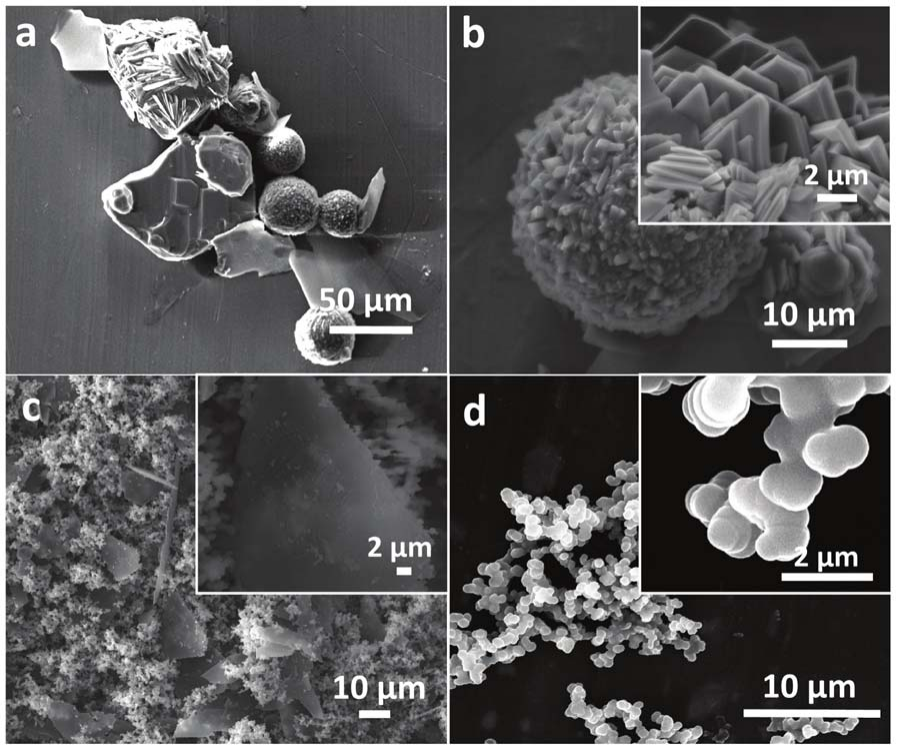
SEM images of silica structures synthesized using histidine in IL [BMIM][BF_4_] involving TEOS to histidine molar ratios of (a and b) 1∶10, (c) 1∶1, and (d) 1∶0.2 respectively. The insets show the higher magnification images of the structures shown in the corresponding main figure.

Similar to lysine and arginine, TEOS hydrolysis in [BMIM][BF_4_] by using relatively large quantity of cationic amino acid histidine (10 mM – 1∶10 TEOS to histidine ratio) resulted in a control over silica morphology, however leads to a completely different set of unique silica structures that contained a mixture of silica microglobules of 20–50 µm diameter and silica plates (Figure 4a–b). Higher magnification SEM imaging of silica microglobules revealed a rough surface entirely comprised of sharp-edged triangular and hexagonal platelets of 1–3 µm edge length and 100–300 nm thickness (Figure 4b). Similar to the previous two cases (lysine and arginine), triangular platelets formed using histidine-mediated hydrolysis of TEOS showed some degree of resemble with triangle-shaped diatom species (e.g. *Trigonium arcticum*) found in nature (Figure 2c), in terms of general shape [7]. However, other typical features of diatomaceous species such as hollow interiors, size and surface patterns were not found to be replicated under the experimental conditions used in this study. At reduced histidine concentration of 1 mM (1∶1 ratio), a mixture of triangular/prismatic silica plates and globular nanospheres were formed (Figure 4c). Further reducing histidine concentration to 0.2 mM (1∶0.2 ratio, Figure 4d) resulted in a complete loss of flat plate-like morphology, and instead resulted in an assembled networks of ca. 800 nm silica spheres.

**Figure 5:**
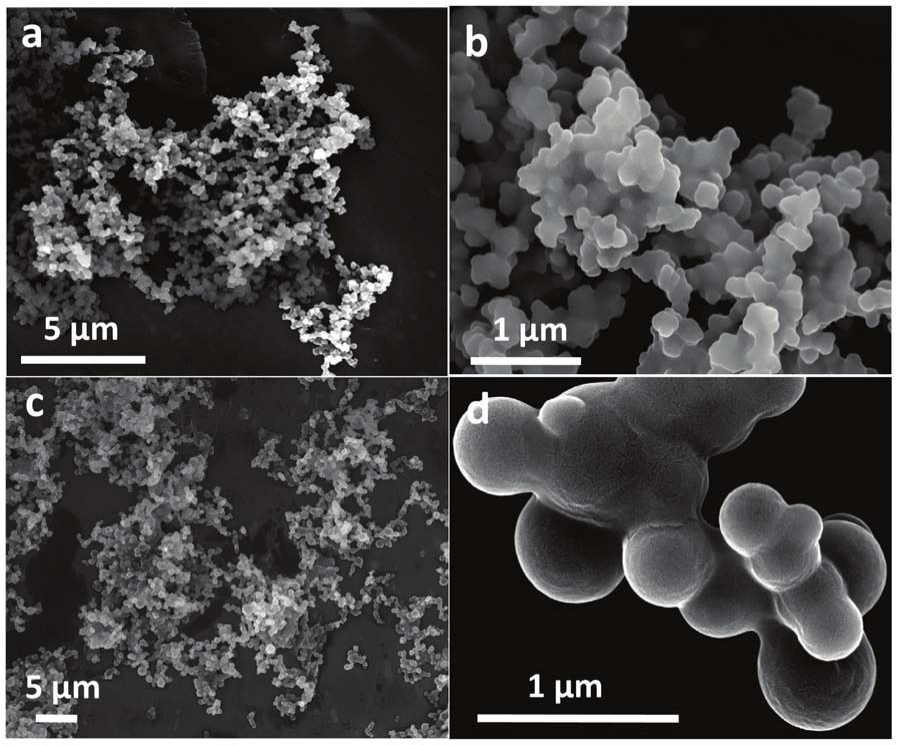
SEM images of silica structures synthesized by hydrolysis of 1 mM TEOS using 10 mM equivalent of (a–b) ammonia, and (c–d) L-serine amino acid in ionic liquid [BMIM][BF_4_].

It is interesting that previous studies in water found that cationic amino acids lysine, arginine and histidine were incapable of promoting TEOS hydrolysis [11], [12], which is clearly not the case in [BMIM][BF_4_] wherein we observe cationic amino acids-mediated TEOS hydrolysis, leading to formation of unique silica structures. Notably, in water, these cationic amino acids were able to induce tetramethylorthosilicate (TMOS) hydrolysis, however only granular silica nanoparticles without any formation of silica superstructures were obtained [11], [12]. These observations point towards the important role that ILs can play in amino acids-mediated morphological control of biomimetic reactions by means of controlling the physico-chemical growth environment via promoting self-assembly/self-organization process. Although the specificity and role of cationic amino acids as morphology-directing agents is evident from these experiments, to unequivocally assign this morphology control to respective cationic amino acids, two additional control experiments were performed. In the first control experiment, 1 mM TEOS was hydrolysed in IL using 10 mM equivalent of liquid ammonia. During ammonia-mediated hydrolysis of TEOS in IL, we did not notice any morphology control, and only agglomerated silica particles were observed (Figure 5a–b). In another control experiment, 1 mM TEOS was hydrolysed in IL using 10 mM equivalent of L-serine amino acid. Serine was chosen as a control because although it is not a cationic amino acid under physiological conditions (isoelectric point ∼5.68), it is present in relatively large proportions along with cationic amino acids lysine, arginine and histidine in silaffin peptides [39]. Similar to the control experiment involving ammonia, serine-mediated hydrolysis of TEOS precursor did not result in a control over silica morphology, and only agglomerated submicron silica particles were formed (Figure 5c–d). These control experiments further confirm the role of cationic amino acids in presence of IL [BMIM][BF_4_] towards biomimetic formation of exquisite diatom-like silica morphologies.

**Figure 6:**
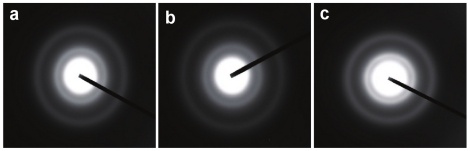
Selected area electron diffraction (SAED) patterns obtained from silica superstructures obtained in ionic liquid [BMIM][BF_4_] by hydrolysis of 1 mM TEOS using 10 mM of cationic amino acids (a) L-lysine, (b) L-arginine, and (c) L-histidine respectively. The diffused ring patterns are either from the TEM grid coated with an amorphous carbon film, and/or from the amorphous silica superstructures.

XRD analysis of the silica structures synthesized by cationic amino acids in [BMIM][BF_4_] further revealed their non-crystalline nature, which is similar to the characteristic amorphous nature of biosilica synthesized by diatoms [3]–[5], [8]–[10]. Additionally, selected area electron diffraction (SAED) analysis performed on individual particle ensembles showed diffused ring patterns typical for amorphous materials, reiterating the amorphous nature of silica superstructures formed in IL (Figure 6).

**Figure 7:**
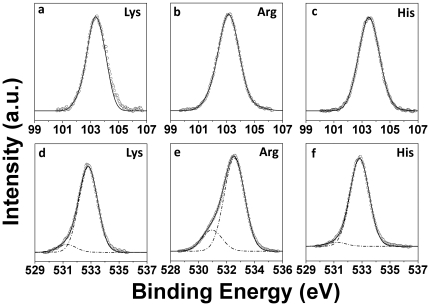
XPS spectra showing Si 2p (a–c) and O 1s (d–f) core levels arising from SiO2 structures synthesized using 10 mM of lysine (a, d); arginine (b, e); and histidine (c, f) respectively.

Since SEM, XRD or SAED analysis did not provide any information about composition of structures seen under SEM, a surface chemical analysis of structures formed using lysine, arginine and histidine was performed using x-ray photoelectron spectroscopy (XPS), which is a highly surface sensitive technique. Figure 7 shows Si 2p and O 1s core level XPS spectra arising from structures obtained from hydrolysis of 1 mM TEOS using 10 mM of respective amino acids. All the XPS spectra have been background corrected using Shirley algorithm, and their respective binding energy (BEs) have been aligned to the C 1s BE of 285 eV [19]–[20]. Si 2p core levels in all the three systems (lysine, arginine and histidine) showed a BE maxima at ca. 103.2±0.2 eV, which corroborates well with previously reported values for SiO_2_ system in an Si-O-Si environment [40]. O 1s core levels in all the three systems could be deconvoluted into two major components with a lower BE component at ca. 531.2±0.1 eV and a higher BE component at ca. 532.6±0.2 eV. The predominant O 1s core level signature at 532.6 eV is due to O atoms bonded in the Si-O-Si network of SiO_2_ particles [40], which one would expect from a predominant SiO_2_ based system. The presence of a minor O 1s low energy BE component at 531.2 eV in each case is quite interesting and is typical of amino acids [41], which can be assigned to free –COO groups present in lysine, arginine and histidine molecules. Presence of –COO signals in these SiO_2_ structures further indicates direct involvement of amino acids towards formation of unique silica morphologies.

In addition to Si 2p and O 1s core levels, we also obtained C 1s and N 1s core level XPS signatures from respective samples, which, on analysis, further suggested incorporation of amine, guanidinium, and imidazolium groups in SiO_2_ structures obtained using lysine, arginine and histidine respectively (data not shown for brevity). However C 1s and N 1s core levels were found to be quite complicated due to interference from imidazolium groups of IL [BMIM][BF_4_], and need to be further investigated through synchrotron-based high energy XPS studies. XPS analysis therefore strongly suggests that novel silica morphologies obtained through a biomimetic route reported here are in fact hybrid organic-inorganic composite biosilica materials.

In combination with XPS data, we are currently investigating the modelling and nuclear magnetic resonance (NMR) aspects of this study, which, in future, might provide a significant understanding of how different amino acids in IL lead to silica superstructures similar to those formed by different diatom species in their marine habitats. How these morphologies are controlled during biomimetic silicification in IL remains an open question at this stage. However, it is well known that in diatoms where similar amorphous hybrid bioinorganic structures are produced, cationic polypeptides play a major role by way of providing a facile template as well as playing the biocatalyst role for silicic acid hydrolysis [10]–[12]. It has been previously established that the conformation and orientation of cationically charged macromolecules in an amino acid matrix formed due to supersaturation and ionicity of the reaction can act as templates for the nucleation and growth of silica structures [27]. Also, parameters like pH, temperature, precursor salt, external ionic strength are known to have significant effect on rate of reaction, oligomerisation of silica, and also towards formation of silica structures [27], [42]. Amino acids concentration dependence on silica morphologies suggests that high amino acid concentrations are responsible for directing growth of complex silica morphologies. In this study, all amino acid solutions used at higher concentrations (10 mM) are close to high degree of super saturation, and the presence of a viscous solvent like ionic liquid may lead to ordered and controlled aggregation of amino acids induced self-organization. This route has been shown to produce complex and hierarchical structures following a diffusion limited aggregation (DLA) process [43], which is also believed to occur during diatomaceous silicification [7], [10], [28].

The specificity of amino acids towards attaining unique silica morphologies in this study also suggests that the diversity in biosilica morphology seen in different diatom species might possibly be due to the compositional differences in cationic polypeptides responsible for biosilicification, wherein lysine, histidine and arginine have been found to be present in different proportions [28], [38]. Detailed studies involving a mixture of amino acids and various polycationic peptides in ILs are currently underway to understand the factors leading to diversity in diatom morphology. Nevertheless, the formation of exquisite diatom-like structures by amino acids-mediated biomimetic silicification in ionic liquids is both intriguing and exciting, as it may lay the foundation of significant new future insights on biosilicification processes occurring in natural habitats. It is expected that this study will fuel wide interest in using ionic liquids as new designer reaction media to study biosilicification and other biomineralization processes, wherein different combination of amino acids, peptides and proteins will be utilized in combination with different ionic liquids to study such natural phenomena.

## Materials and Methods

### Materials

1-butyl-3-methylimidazolium tetrafluoroborate ([BMIM-BF_4_]) was purchased from Ionic Liquid Technologies (IoLiTec). Amino acids L-lysine, L-arginine, L-histidine, and L-serine, as well as tetraethylorthosilicate (TEOS) were purchased from Sigma-Aldrich. All chemicals were used as received.

### Biosilica formation using cationic amino acids

In a typical synthesis of silica structures, 1 M, 100 mM and 20 mM stock solutions of three cationic amino acids (L-lysine, L-arginine and L-histidine) and non-cationic amino acid L-serine were separately prepared in MilliQ deionized water. 10 µL of respective amino acid stock solutions were added to 490 µL of IL [BMIM][BF_4_]. To these 500 µL amino acids in IL, 500 µL of ionic liquid containing 2 mM tetraethylorthosilicate (TEOS) was added. This resulted in 1 mL reactions containing a final TEOS concentration of 1 mM and final amino acids concentrations of 10 mM, 1 mM and 0.2 mM for respective amino acids. Therefore, final 1 mL reaction volume contained only 10 µL water and 1 µL TEOS, while retaining remaining 989 µL IL. Water and TEOS volumes were kept to the minimum possible levels to avoid a potential change in the properties of ILs with admixtures. In a control experiment, 10 µL of deionized water (without amino acid) was used in a 1 mL reaction containing 1 mM TEOS. In another control experiment, amino acids were replaced with liquid ammonia while maintaining the final ammonia concentration as 10 mM. The reactions were pursued for 16 h at 25±0.1°C under non-stirring conditions, during which all the reactions involving amino acids or ammonia became turbid. However, no turbidity was observed in the control reaction containing water, thereby negating the possibility of water-mediated hydrolysis of TEOS in [BMIM][BF_4_] and suggesting the role of cationic amino acids towards TEOS hydrolysis in [BMIM][BF_4_]. The reaction products in IL were further centrifuged at 8,000 rpm for 10 min and thoroughly washed with acetonitrile to remove viscous IL for further characterisation.

### Materials Characterisation

Samples for Scanning Electron Microscopy (SEM) were prepared by drop casting the sample onto an aluminium stub and coated with platinum prior to imaging to minimize surface charging. SEM images were obtained using FEI Nova NanoSEM instrument operated at an accelerating voltage of 30 kV. X-ray diffraction (XRD) measurements were carried out on a Bruker D8 Advance XRD instrument operated at a voltage of 50 kV and a current of 35 mA with Cu Kα radiation. Samples for selected area electron diffraction (SAED) analysis were primed by drop casting sonicated samples on a carbon coated copper grid. SAED was performed using JEOL 1010 TEM instrument operated at an accelerating voltage of 100 kV. For X-ray photoemission spectroscopy (XPS), samples were prepared by drop casting the sample on a 100 nm evaporated thin film coated Au substrates, and measurements were carried out using Thermo K-Alpha XPS instrument at a pressure better than 1×10–9 Torr (1 Torr  = 1.333×102 Pa). The general scan and C 1s, Si 2p, O 1s, and N 1s core level spectra for the samples were recorded with un-monochromatized Mg Kα radiation (photon energy of 1253.6 eV) at a pass energy of 20 eV and electron take off angle of 90°. The overall resolution was 0.1 eV for XPS measurements. The core level spectra were background corrected using Shirley algorithm and chemically distinct species were resolved using a nonlinear least squares fitting procedure. The core level binding energies (BE) were aligned with adventitious carbon binding energy of 285 eV.
